# Detection of a peritumoral pseudocapsule in patients with renal cell carcinoma undergoing robot-assisted partial nephrectomy using enhanced MDCT

**DOI:** 10.1038/s41598-021-81922-0

**Published:** 2021-01-26

**Authors:** Makoto Toguchi, Toshio Takagi, Yuko Ogawa, Satoru Morita, Kazuhiko Yoshida, Tsunenori Kondo, Junpei Iizuka, Hideki Ishida, Yoji Nagashima, Kazunari Tanabe

**Affiliations:** 1 Department of Urology, Tokyo Women’s Medical University, 8-1, Kawada-cho, Shinjuku-ku, Tokyo, 162-8666 Japan; 2 Department of Diagnostic Imaging and Nuclear Medicine, Tokyo Women’s Medical University, Tokyo, Japan; 3 Department of Urology, Tokyo Women’s Medical University Medical Center East, Tokyo, Japan; 4 Department of Surgical Pathology, Tokyo Women’s Medical University, Tokyo, Japan

**Keywords:** Oncology, Urology

## Abstract

To investigate the detection of peritumoral pseudocapsule (PC) using multi-detector row computed tomography (MDCT) for tumors resected by robot-assisted laparoscopic partial nephrectomy (RAPN) for T1 renal cell carcinoma (RCC). Study participants included 206 patients with clinical T1 RCC who underwent RAPN between October 2017 and February 2018. Two radiologists who were blinded to the pathological findings evaluated the computed tomography (CT) images. Radiological diagnosis of a PC was defined by a combination of observations, including a low-attenuation rim between the tumor and renal cortex in the cortico-medullary phase and a high-attenuation rim at the edge of the tumor in the nephrogenic or excretory phase. A PC was detected on CT in 156/206 tumors (76%) and identified by pathology in 182/206 (88%) tumors including 153/166 (92%) clear cell RCC, 13/14 (93%) papillary RCC, and 7/16 (44%) chromophobe RCC. In the whole cohort, CT findings showed a sensitivity of 81.3% (148/182), specificity of 66.7% (16/24), and positive predictive value of 94.9% (148/156). When the data were stratified according to pathological subtypes, MDCT was observed to have a sensitivity of 86.9% (133/153) and specificity of 61.5% (8/13) in clear cell RCC, sensitivity of 38.5% (5/13) and specificity of 100% (1/1) in papillary RCC, and sensitivity of 44.4% (4/7) and specificity of 66.7% (6/9) in chromophobe RCC. A low or high-attenuation rim around the tumor in the cortico-medullary or nephrographic-to-excretory phase indicates a PC of RCC, though the accuracy is not satisfactory even with 64- or 320-detector MDCT.

## Introduction

The purpose of partial nephrectomy (PN) for renal tumors is to preserve renal function while securing cancer control and minimizing complications. An important factor for preserving renal function is maximal normal parenchymal preservation, which is achieved by precise resection and reconstruction. Robot-assisted partial nephrectomy (RAPN) realizes precision with a stable view and wide range operability and is indicated for more complex tumors. To achieve maximal normal parenchymal preservation, tumor enucleation, which removes the tumor along with the peritumoral pseudocapsule (PC), could be an ideal surgical method^1,2^. The feasibility and efficacy of this technique were evaluated in several studies^3–6^.

The presence of peritumoral PC is dependent on pathological subtype or tumor characteristics. In our previous study, a peritumoral PC was observed in 92% of clear cell renal cell carcinoma (RCC) cases, 89% of papillary RCC cases, and 86% of clear cell papillary RCC cases. In contrast, chromophobe RCC was surrounded by PC in only 50% of cases^7^. In addition, small tumors tended to lack PC^7^. When resecting tumors, greater caution is warranted for tumors without PC, such as chromophobe RCC or small tumors, to avoid damaging the tumor surface. Therefore, determining the presence or absence of PC before surgery is useful for delineating resection technique, including standard PN or enucleation. Past reports have evaluated PC of RCC using magnetic resonance imaging (MRI) and computed tomography (CT) in a small number of patients^8–10^. Compared to CT, T2-weighted MRI is more sensitive for the detection of PC of RCC^9^. However, MRI is not routinely performed for preoperative evaluation of RCC. On the other hand, MDCT is performed in most patients to radiologically diagnose malignancy, except for those with allergy to the dye agent or with severe chronic disease. A detailed evaluation using multi-detector low computed tomography (MDCT) with more than 64 detectors including thin slice images in a large number of patients has not been performed.

The aim of this study was to compare the peritumoral PC status between preoperative enhanced MDCT and pathological findings and determine whether a PC was present by evaluating preoperative enhanced MDCT thin slice images in a relatively large number of patients.

## Patients and methods

### Patient population

This study was an institutional review board-approved (Institutional Review Board approval no. 4720) retrospective review of consecutive patients who underwent RAPN at a single institution between January 2015 and February 2018. Patients without preoperative enhanced CT and with benign pathological diagnosis were excluded. From August 2016 to February 2018, 249 patients underwent RAPN for RCC at a single institution. A total of 43 patients were excluded because preoperative contrast-enhanced CT was not performed in our institution. The final study group comprised 206 patients. Tumor complexity was determined according to the R.E.N.A.L nephrometry score (RENAL-NS)^11^. Tumor stage was determined according to the 2009 TNM classification^12^, and pathological diagnosis was established according to the 2016 World Health Organization classification^13^. In the current study, a PC was defined as present if it completely surrounded the tumor. All methods were carried out in accordance with relevant guidelines and regulations. All experimental protocols were approved by a Tokyo Women’s Medical University institutional and/or licensing committee. Informed consent was obtained from all subjects or, if subjects were aged under 18 years, from a parent and/or legal guardian.

### CT examinations

Images were obtained using 64-row detector scanners (Aquilion 64; Canon Medical Systems, Otawara, Japan) or 320-row detector scanners (Aquilion One; Canon Medical Systems) at the following settings: a 1:1 table pitch; collimation, 0.5–1 mm; reconstruction thickness/interval, 1.0 mm/1.0 mm; 100–120 kVp with Auto mA; and adaptive iterative dose reduction (AIDR)^14^. Corticomedullary phase images were obtained using a bolus-tracking contrast monitoring system after injecting contrast material for 30 s. The nephrographic and excretory phase images were subsequently obtained 90 and 300 s after injecting contrast medium. The total iodine dose was 600 mg/kg body weight (maximum dose: 45 g and maximum injection rate: 5.0 mL/s).

### CT evaluation

**Figure 1:**
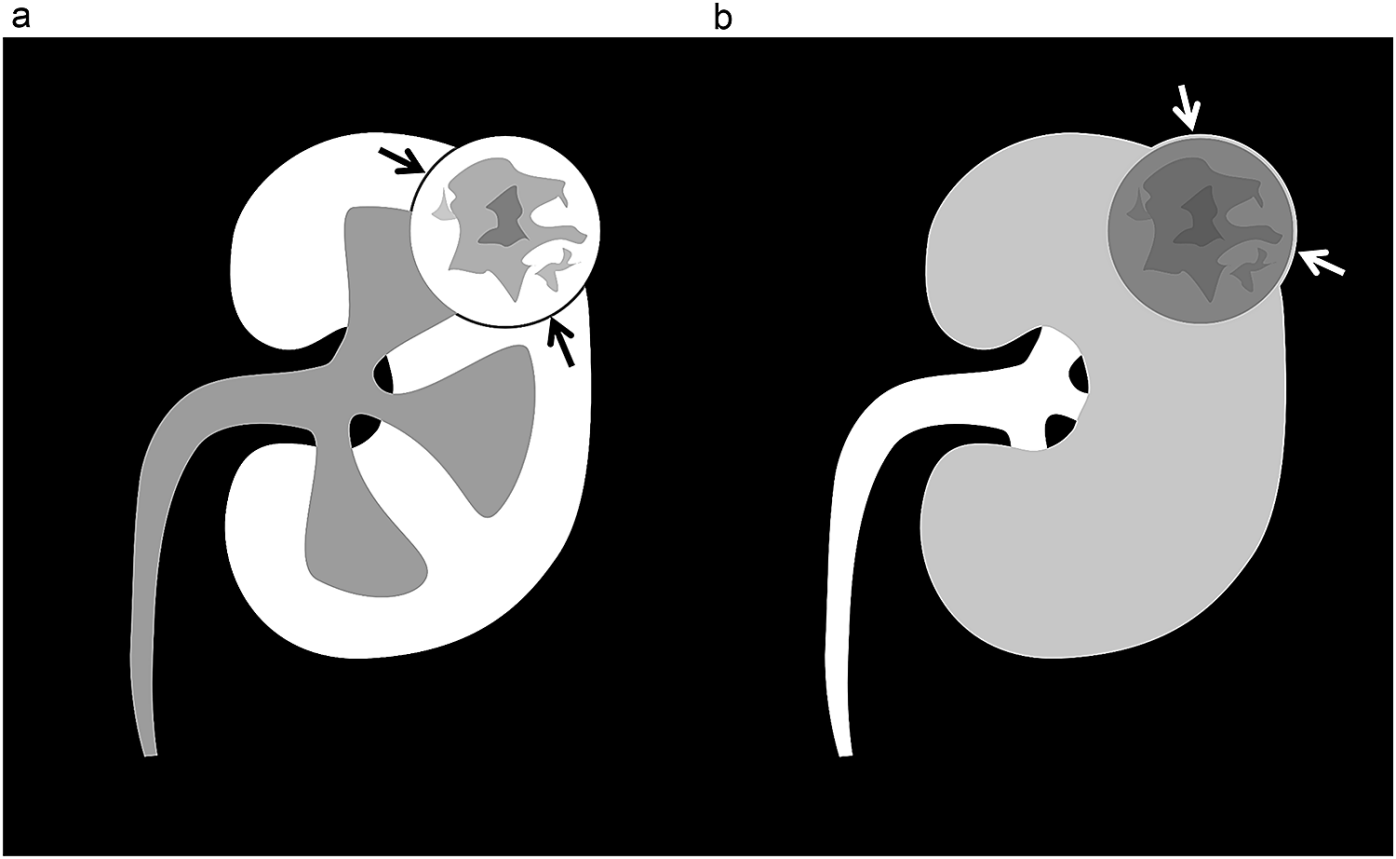
Schematic images of a peritumoral pseudocapsule on contrast-enhanced computed tomography images: a thin low-attenuation rim between the tumor and renal cortex (black arrows) on the cortico-medullary phase (**a**) and a higher-attenuation rim at the edge of the tumor (white arrows) on the nephrographic or excretory phase (**b**).

**Figure 2:**
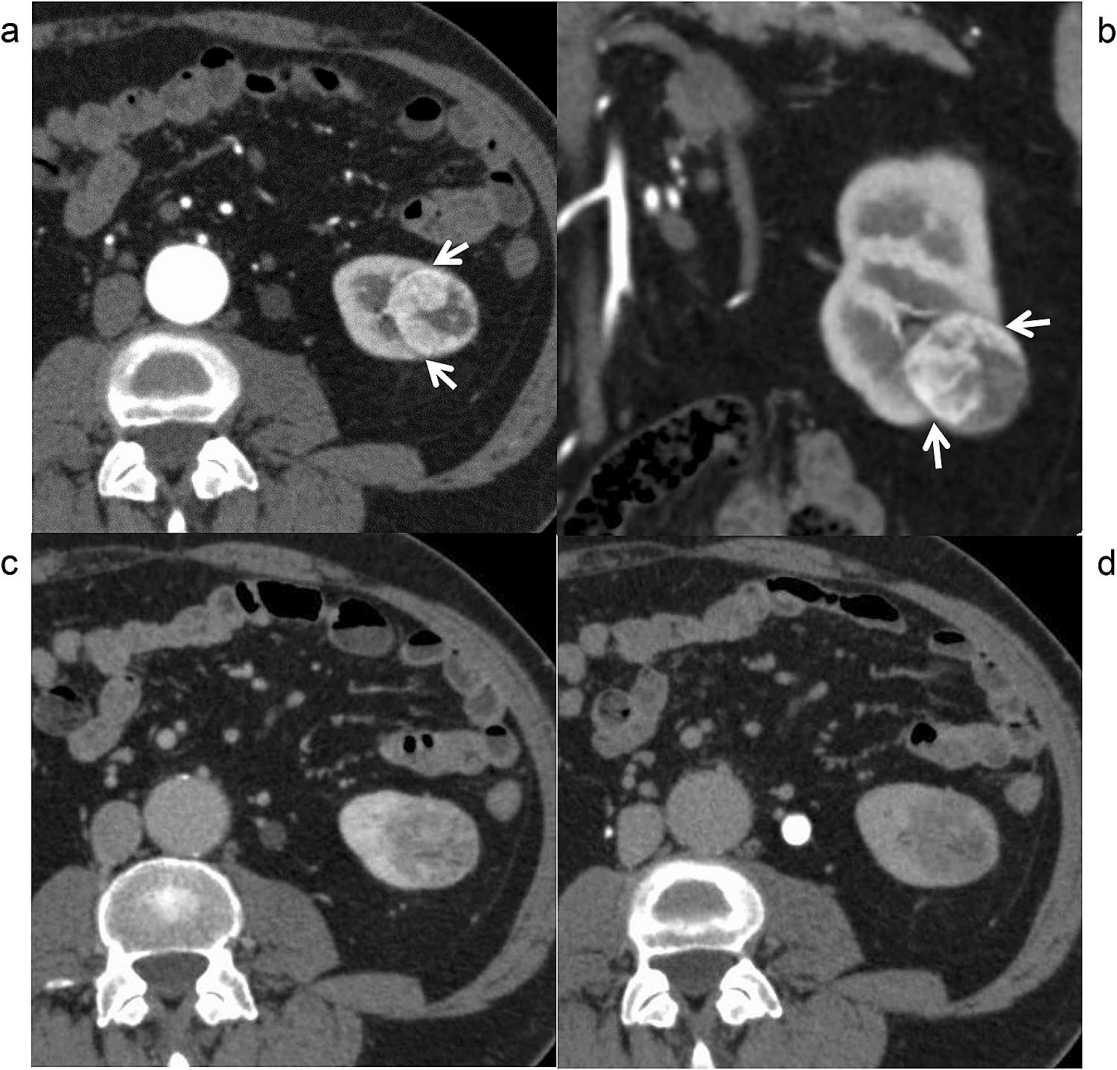
Contrast-enhanced computed tomography of a clear cell renal cell carcinoma with a pathological peritumoral pseudocapsule in a 57-year-old man. A thin low-attenuation rim is observed between the tumor and renal cortex (arrows) on the cortico-medullary phase trans-axial (**a**) and coronal images (**b**). A high-attenuation rim is not observed on the nephrographic (**c**) or excretory phase (**d**) images.

**Figure 3:**
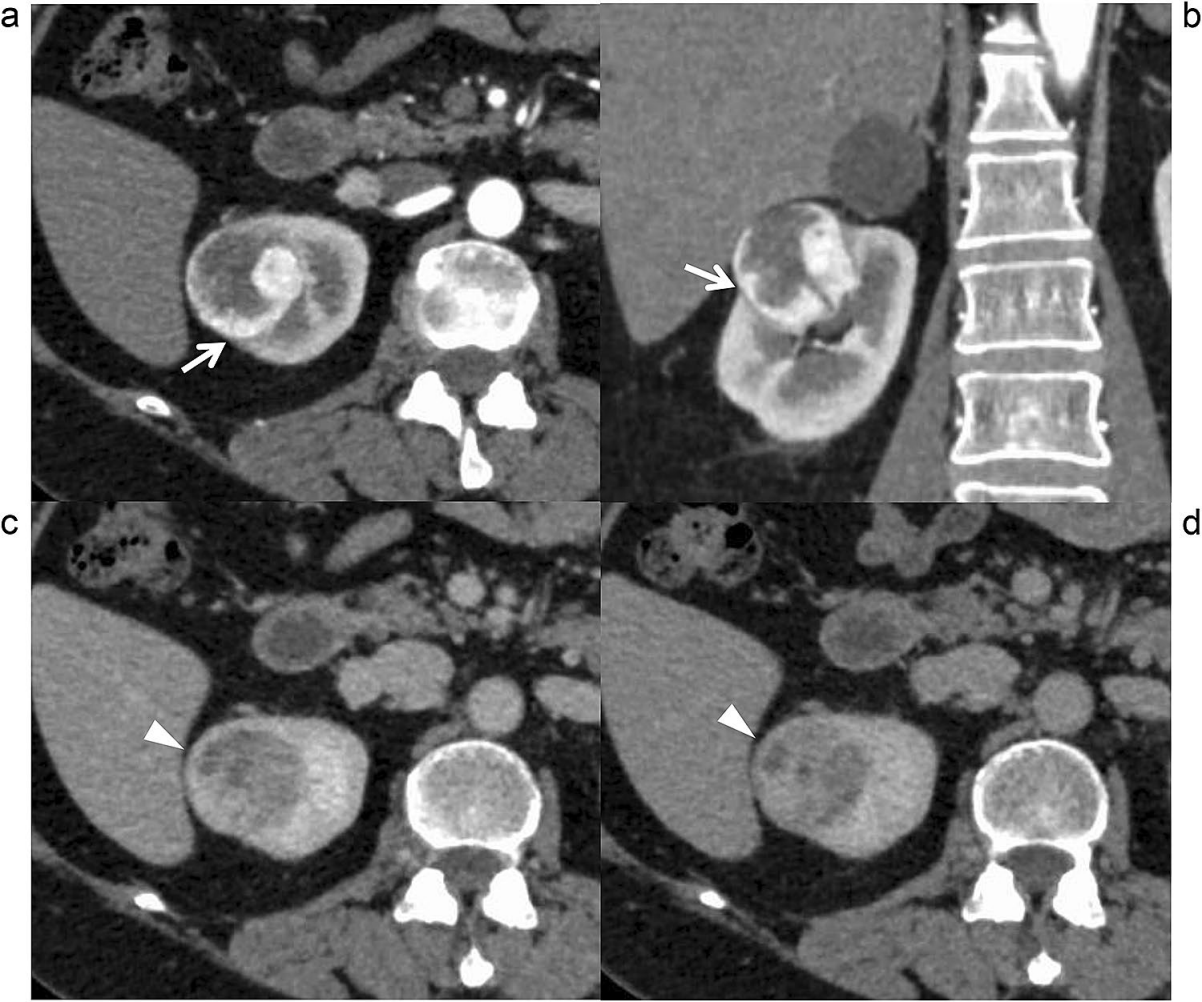
Contrast-enhanced computed tomography of a clear cell renal cell carcinoma without a pathological peritumoral pseudocapsule in a 53-year-old women. A thin low-attenuation rim is observed partly between the tumor and renal cortex (arrows) on the cortico-medullary phase trans-axial (**a**) and coronal images (**b**). A suspected high-attenuation rim is observed on the nephrographic (**c**) or excretory phase (**d**) images. These findings represent false-positive results of a pseudocapsule.

Images were independently evaluated by an experienced radiologist and a radiology resident (A and B) using a viewer (ShadeQuest/ViewR; Yokogawa Medical Solutions, Tokyo, Japan) with a three-dimensional workstation (Aquarius iNtuition, TeraRecon, Foster City, CA), and a final diagnosis was determined after discussion. First, thin slice images with 1.0-mm thickness and an interval on the trans-axial plane were evaluated. If required, multi-planer reconstruction (MPR) images at various angles were reconstructed and evaluated using the workstation. All images were randomized, and the reviewers were blinded to information regarding PC. Detection of the PC was assessed using a 2-point scale system as follows: 1, absent; 2, present. A peritumoral PC was defined as present if thin low-attenuation rim was detected between the tumor and renal cortex on corticomedullary phase images (Figs. 1a, 2a,b, 3a,b). If a higher attenuating rim at the edge of the tumor compared to tumor enhancement was detected on the nephrographic or excretory phase images (Figs. 1b, 3c,d), it was also defined as present. Figure 2c,d showed radiologically lack of peritumoral PC on the nephrographic and excretory phase images. Where discrepancies occurred between the reviewers’ scores, a definitive score was obtained by consensus. Scores of 1 and 2 were categorized as negative, and scores of 3 and 4 as positive for the absence or presence of PC. The sensitivity, specificity, positive predictive value, and negative predictive value for detecting PC on each phase were calculated using histopathologic data as the standard of reference.

### Surgery

Three surgeons performed transperitoneal RAPN as previously described^15^. Briefly, an arterial clamp with warm ischemia, three robotic arms, and two or three assistant ports were used. After tumor excision, the tumor bed was sutured using the running technique, and the renal artery was unclamped. Arterial hemostasis was confirmed, and the renal parenchyma was closed using a barbed suture. Retroperitoneal RAPN was performed using three robotic arms and one assistant port. The arterial clamping and renorrhaphy techniques were similar to those used in the transperitoneal approach.

### Statistical analysis

All statistical analyses were performed using JMP v. 11.2.0 (SAS Institute Inc., Cary, NC, USA). Continuous variables were analyzed with the Mann–Whitney U test, and categorical variables were analyzed with the χ^2^ test. *P* values < 0.05 were considered statistically significant. The interobserver variability was estimated using Cohen’s kappa test. The κ values indicated poor (< 0.40), moderate (0.41–0.60), good (0.61–0.80), and excellent (0.81–1.00) agreements.

### Ethical approval

All procedures involving human participants were in accordance with the ethical standards of the institutional and/or national research committee and 1964 Helsinki declaration and its later amendments or comparable ethical standards (Institutional Review Board approval no. 4720).

## Results

Patient characteristics are shown in Table 1. Of the 206 patients, 160 (78%) were male. The median age was 58 years, and the median BMI was 24 kg/m^2^. The ASA score was 1 in 38 patients (18%), 2 in 146 patients (71%), and 3 in 22 patients (11%). The median tumor size was 28 mm, and clinical T1 was observed in 166 patients (81%). Low, intermediate, and high complexity tumors were observed in 39%, 51%, and 10% of patients, respectively. The enucleation technique was performed in 37% (77) of patients in accordance with the surface-intermediate-base score^16^. The median operation time and median warm ischemia time was 154 min and 16 min, respectively. A positive surgical margin was observed in five patients (2%). The median postoperative length of hospital stay was 3 days. Pathological subtype was clear cell type in 166 (81%) patients, papillary type in 14 (7%) patients, chromophobe in 16 (8%) patients, and others in 10 (5%) patients. Fuhrman grade was 1 in 14%, 2 in 66%, 3 in 19%, and 4 in 1% of patients. Tumor recurrence was not found during the study period.

**Table 1:** Characteristics of patients.

Number of patients, n	206
**Patient characteristics**	
Male, n	160 (78%)
Age, years, median (IQR)	58 (47–67)
BMI, kg/m^2^, median (IQR)	24 (22–27)
*ASA score, n*	
1	38 (18%)
2	146 (71%)
3	22 (11%)
**Tumor characteristics**	
Tumor size, mm, median (IQR)	28 (19–39)
*Clinical T stage, n*	
T1a	166 (81%)
T1b	40 (19%)
*Tumor complexity, n*	
Low	81 (39%)
Intermediate	105 (51%)
High	20 (10%)
**Resection technique, n**	
Enucleation	77 (37%)
Resection	129 (63%)
**Perioperative outcomes**	
Operation time, min, median (IQR)	154 (132–181)
WIT, min, median (IQR)	16 (13–21)
EBL, ml, median (IQR)	50 (20–81)
PSM, n	5 (2.4%)
PLOS, days, median (IQR) PLOS, days, median (IQR)	3 (3–4)
**Pathological characteristics**	
*Subtype, n*	
Clear cell type	166 (81%)
Papillary type	14 (7%)
Chromophobe type	16 (8%)
Others, n	10 (5%)
**Fuhrman grade, n**	
1	28 (14%)
2	136 (66%)
3	39 (19%)
4	3 (1%)

*BMI* body mass index, *IQR* interquartile range, *ASA* American Society Anesthesiologists, *WIT* warm ischemia time, *EBL* estimated blood loss, *PSM* positive surgical margin, *PLOS* postoperative length of hospital stay.

In total, peritumoral PC was pathologically observed in 88% (182/206) of patients. MDCT detected PC in 76% (156/206) of tumors. Table 2 shows the detection of PC by CT imaging. For the cortico-medullary phase, sensitivity and specificity were 65.6% and 66.7%, respectively. A similar sensitivity (23–26%) and specificity (87.5%) were observed in both the nephrogenic phase and excretory phase. For total evaluation, sensitivity and specificity were 81.3% and 66.7%, respectively. In addition, positive predictive value and negative predictive value were 94.6% and 32.0%, respectively. The κ value showed moderate to good agreement between two observers (κ value = 0.55 in the early phase, 0.82 in the nephrographic phase, 0.74 in the excretory phase, and 0.60 in total).

**Table 2:** Detection of peritumoral pseudocapsule on each phase imaging of contrast enhanced CT.

	In total	Corticomedullary phase	Nephrographic phase	Excretory phase
Sensitivity	81.3 (148/182)	65.4% (119/182)	25.8% (47/182)	30.0% (42/140)
Specificity	66.7% (16/24)	66.7% (16/24)	87.5% (21/24)	87.5% (21/24)
PPV	94.9% (148/156)	93.7% (119/127)	94.0% (47/50)	93.3% (42/45)
NPV	32.0% (16/50)	20.3% (16/79)	13.5% (21/156)	13.0% (21/161)

*PPV* positive predictive value, *NPV* negative predictive value.

Table 3 provides detection of PC according to pathological subtype. For clear cell RCC, sensitivity and specificity were 86.9% and 61.5%, respectively. Regarding other subtypes, sensitivity and specificity were 57.1% and 66.7% for chromophobe RCC, and 38.5% and 100% for papillary RCC.

**Table 3:** Detection of peritumoral pseudocapsule in each pathological subtype on contrast enhanced CT.

	Clear cell (n = 166)	Chromophobe (n = 16)	Papillary (n = 14)
Sensitivity	86.9% (133/153)	57.1% (4/7)	38.5% (5/13)
Specificity	61.5% (8/13)	66.7% (6/9)	100% (1/1)
PPV	96.4% (133/138)	57.1% (4/7)	100% (5/5)
NPV	28.6% (8/28)	66.7% (6/9)	11.1% (1/9)

*PPV* positive predictive value, *NPV* negative predictive value.

## Discussion

Our study demonstrated a high positive predictive value of a low- or high-attenuation rim around the tumor in the cortico-medullary or nephrogenic-to-excretory phase for detecting PC by evaluating preoperative enhanced thin-slice MDCT images in a relatively large number of patients; however, the diagnosis of PC of RCC with MDCT is still limited because of the low negative predictive value.

Following the introduction of robotic surgery, complex renal tumors could be subjected to partial nephrectomy, and more sophisticated resection techniques were required. For large renal hilum tumors abutting renal vessels or the collecting system, the enucleation technique is applied in RAPN to avoid damaging these structures. The peritumoral PC is a landmark for the resection layer for enucleation; therefore, awareness of the presence or absence of PC before surgery is useful for surgeons to perform optimal RAPN for complicated tumors. Although several articles have addressed the detection of peritumoral PC using CT^8^ or MRI^9,10,17^, our study provides novel insight, as we assessed a large number of tumors and various pathological subtypes.

PC formation is the result of tumor growth which causes compression, ischemia, and necrosis of the adjacent renal parenchyma and results in deposition of fibrous tissue^8^. This fibrous tissue may be detected as a thin linear regular hypointense band on MRI T2- and T1-weighted images surrounding the tumor^10^ or a regular, high-, or low-attenuation rim surrounding renal neoplasm on MDCT^8^. In our study, the definition of PC was a low-attenuation rim between the tumor and renal cortex on corticomedullary phase images or high-attenuation rim at the edge of the tumor compared to tumor enhancement on the nephrographic or excretory phase images. Notably, the low-attenuation rim between the tumor and renal cortex on corticomedullary phase images, which is detected on thin-slice, multi-planar reconstruction images taken at various angles, is particular to our study. By combining these several phase images, we achieved highly accurate detection of PC using a 64-row detector scanner or a 320-row detector scanner MDCT.

A previous study reported the detection of PC in RCC with MDCT, despite a small sample size^8^. In their study, MDCT detected PC in 69% (20/29) of RCCs, and pathologically confirmed PC was observed in 83% (24/29) of RCCs, showing high sensitivity (83%), high specificity (80%), and high positive predictive value (95%). The definition of PC in their study was described as the presence of a regular, high- or low-attenuation rim surrounding renal neoplasm^8^. In our study, PC was pathologically observed in 88% (182/206) and radiologically diagnosed in 76% (156/206), showing high sensitivity (81.3%), high specificity (66.7%), and high positive predictive value (94.9%). Although several differences exist among these studies such as the definition of PC by radiological examination and number of patients included, the detection of PC by CT is generally considered to be accurate.

Other imaging modality such as MRI showed a high accuracy of detection of pseudocapsule. In one study, a PC was detected in 48% of RCC cases (26 of 54) based on pathological findings and in 50% of RCC cases (26 of 52) by MRI; on MRI, the pseudocapsule was defined as a thin linear regular hypointense band on T2- and T1-weighted images surrounding the tumor, with a high sensitivity (67.9%), high specificity (90.9%), and high accuracy (74.4%)^9^. In advance, Papalia et al. reported accuracy of MRI to identify pseudocapsule invasion in renal tumors. They classified the pseudocapsule patterns as follows: absence of PC, presence of a clearly identifiable PC, focally interrupted PC, and clearly interrupted and infiltrated PC. With either pattern, high sensitivity (77–98%) and high specificity (83–96%) were achieved^18^.

The present study demonstrated high sensitivity, specificity, and positive predictive value for detection of peritumoral PC by enhanced MDCT in the total cohort. On the other hand, the detection rate was different among pathological subtypes as shown in Table 3. For clear cell RCC, PC was observed in 83.1% (138/166) by CT and 92.2% (153/166) by pathological diagnosis, showing high sensitivity (86.9%) and positive predictive value (96.4%) and moderate specificity (61.5%). For chromophobe RCC, PC was observed in 43.8% (7/16) by CT and 43.8% (7/16) by pathological diagnosis, showing moderate sensitivity (57.1%), specificity (66.7%), and positive predictive value (57.1%). For papillary RCC, PC was detected in 35.7% (5/14) by CT and 92.9% (3/14) by pathological diagnosis, showing low sensitivity (38.5%), high specificity (100%), and positive predictive value (100%). These different results may be associated with an enhanced pattern according to pathological subtypes. Clear cell RCC tended to be strongly enhanced at the corticomedullary phase, and the dye agent was washed out at the nephrographic and expiratory phases. The low-attenuation rim between the tumor and renal cortex on corticomedullary phase images, which is one of the definitions of PC in our study, is particular to tumors enhanced in the corticomedullary phase, a characteristic of clear cell RCC. In contrast, chromophobe RCC tended to be moderately and homogeneously enhanced at the corticomedullary phase, and the enhancement slowly waned at the nephrogenic and expiratory phases. These enhanced patterns may cause difficulty in detecting peritumoral PC.

The presence or absence of PC is dependent on several tumoral factors. According to our previous study, small tumors and chromophobe RCC tended to lack PC^7^. In the present study, the presence of peritumoral PC differed among the pathological subtypes. In chromophobe RCC, 41.2% (7/16) of tumors were surrounded by PC. In total, 92.3% (153/166) of tumors in clear cell RCC and 92.9% (13/14) of tumors in papillary RCC contained pathologically confirmed PC, which may also be associated with the differences in MDCT diagnosis of PC.

The present study has several limitations. First, the study was retrospective, performed in a single institution, and included a population of tertiary care patients. Second, in the current study, a PC was defined as present if it completely surrounded the tumor, and tumors with partial presence of PC were excluded, which may have affected the detection rate of PC by MDCT. In addition, pathological findings regarding tumor invasion to the pseudocapsule, which may be related to oncological prognosis^19^, is not described in the present study due to lack of data. The relation between preoperative imaging study and pseudocapsule tumor invasion in pathological diagnosis will be important information for physicians. Third, for the analysis of pathological subtypes, the number of tumors with papillary RCC and chromophobe RCC was small, which may be insufficient to accurately reflect the accuracy for detecting PC. Fourth, relatively small size tumors were included in this study, suggesting that our study does not reflect large size renal tumors. In fact, the distribution of clinical tumor stage included in the study was 81% in T1a and 19% in T1b. In addition, 100% (40/40) of T1b tumors were surrounded by pseudocapsule, but 86% (142/166) of T1a tumors were surrounded by a pseudocapsule. The strengths of this study were its relatively large number of included patients and utilization of fine imaging MDCT such as a 64-row or 320-row detector scanner MDCT. In addition, respective analyses according to pathological subtypes including clear cell RCC, papillary RCC, and chromophobe were performed, which have not been reported to date.

In conclusion, a low- or high-attenuation rim around the tumor in the cortico-medullary or nephrogenic-to-excretory phase has a high positive predictive value, although the diagnosis of PC of RCC with MDCT is still limited because of its low negative predictive value.

## Abbreviations

RAPN: Robot-assisted partial nephrectomy
RP: Retroperitoneal approach
TP: Transperitoneal approach
eGFR: Estimated glomerular filtration rate
RENAL-NS: RENAL nephrometry score
EBL: Estimated blood loss
BMI: Body mass index
PLOS: Postoperative length of hospital stay
WIT: Warm ischemic time

## References

[1] Minervini A, Campi R, Di Maida F, Mari A, Montagnani I, Tellini R (2018). Tumor-parenchyma interface and long-term oncologic outcomes after robotic tumor enucleation for sporadic renal cell carcinoma. Urol. Oncol..

[2] Minervini A, Campi R, Lane BR, De Cobelli O, Sanguedolce F, Hatzichristodoulou G (2020). Impact of resection technique on perioperative outcomes and surgical margins after partial nephrectomy for localized renal masses: A prospective multicenter study. J. Urol..

[3] Longo N, Minervini A, Antonelli A, Bianchi G, Bocciardi AM, Cunico SC (2014). Simple enucleation versus standard partial nephrectomy for clinical T1 renal masses: perioperative outcomes based on a matched-pair comparison of 396 patients (RECORd project). Eur. J. Surg. Oncol..

[4] Minervini A, Ficarra V, Rocco F, Antonelli A, Bertini R, Carmignani G (2011). Simple enucleation is equivalent to traditional partial nephrectomy for renal cell carcinoma: results of a nonrandomized, retrospective, comparative study. J. Urol..

[5] Blackwell RH, Li B, Kozel Z, Zhang Z, Zhao J, Dong W (2016). Functional implications of renal tumor enucleation relative to standard partial nephrectomy. Urology.

[6] Takagi T, Kondo T, Tachibana H, Iizuka J, Omae K, Yoshida K (2017). Comparison of surgical outcomes between resection and enucleation in robot-assisted laparoscopic partial nephrectomy for renal tumors according to the surface-intermediate-base margin score: A Propensity score-matched study. J. Endourol..

[7] Takagi T, Yoshida K, Kondo T, Kobayashi H, Iizuka J, Okumi M (2019). Peritumoral pseudocapsule status according to pathological characteristics from robot-assisted laparoscopic partial nephrectomy for localized renal cell carcinoma. Int. J. Urol..

[8] Tsili AC, Argyropoulou MI, Gousia A, Kalef Ezra J, Sofikitis N, Malamou Mitsi V (2012). Renal cell carcinoma: value of multiphase MDCT with multiplanar reformations in the detection of pseudocapsule. AJR Am. J. Roentgenol..

[9] Yamashita Y, Honda S, Nishiharu T (1996). Detection of pseudocapsule of renal cell carcinoma with MR imaging and CT. Am. J. Roentgenol..

[10] Roy C, El Ghali S, Buy X (2005). Significance of the pseudocapsule on MRI of renal neoplasms and its potential application for local staging: A retrospective study. Am. J. Roentgenol..

[11] Kutikov A, Uzzo RG (2009). The R.E.N.A.L. nephrometry score: A comprehensive standardized system for quantitating renal tumor size, location and depth. J. Urol..

[12] Sobin LH, Compton CC (2010). TNM seventh edition: What's new, what's changed: communication from the International Union Against Cancer and the American Joint Committee on Cancer. Cancer.

[13] Moch H, Cubilla AL, Humphrey PA (2016). The 2016 WHO classification of tumours of the urinary system and male genital organs—Part A: Renal, penile, and testicular tumours. Eur. Urol..

[14] Morita S, Tajima T, Yamazaki H, Sonoyama Y, Nishina Y, Kenji O (2015). Early postoperative screening by contrast-enhanced CT and prophylactic embolization of detected pseudoaneurysms prevents delayed hemorrhage after partial nephrectomy. J. Vasc. Interv. Radiol..

[15] Takagi T, Kondo T, Yoshida K (2018). Comparison of kidney function in the early postoperative period in transperitoneal robot-assisted laparoscopic partial nephrectomy between anterior and posterior renal tumors: A propensity score-matched study. J. Endourol..

[16] Minervini A, Carini M, Uzzo RG, Campi R, Smaldone MC, Kutikov A (2014). Standardized reporting of resection technique during nephron-sparing surgery: The surface-intermediate-base margin score. Eur. Urol..

[17] Van Oostenbrugge TJ, Runneboom W, Bekers E (2019). MRI as a tool to assess surgical margins and pseudocapsule features directly following partial nephrectomy for small renal masses. Eur. Radiol..

[18] Papalia R, Panebianco V, Mastroianni R, Del Monte M, Altobelli E, Faiella E (2020). Accuracy of magnetic resonance imaging to identify pseudocapsule invasion in renal tumors. World J. Urol..

[19] Minervini A, Di Cristofano C, Lapini A, Marchi M, Lanzi F, Giubilei G (2009). Histopathologic analysis of peritumoral pseudocapsule and surgical margin status after tumor enucleation for renal cell carcinoma. Eur. Urol..

